# Adaptive optics visual simulators: a review of recent optical designs and applications [Invited]

**DOI:** 10.1364/BOE.473458

**Published:** 2022-11-17

**Authors:** Susana Marcos, Pablo Artal, David A. Atchison, Karen Hampson, Richard Legras, Linda Lundström, Geunyoung Yoon

**Affiliations:** 1 Center for Visual Sciences; The Institute of Optics and Flaum Eye Institute, University of Rochester, New York 14642, USA; 2 Laboratorio de Optica, Universidad de Murcia, Campus Universitario de Espinardo, 30100, Spain; 3 Centre for Vision and Eye Research, Queensland University of Technology, Brisbane Q, 4059, Australia; 4 Department of Optometry, University of Manchester, Manchester M13 9PL, UK; 5 LuMIn, CNRS, ENS Paris-Saclay, Université Paris-Saclay, CentraleSupelec, Université Paris-Saclay Orsay, 91400, France; 6 KTH (Royal Institute of Technology), Stockholm, 10691, Sweden; 7 College of Optometry, University of Houston, Houston, 77004, USA

## Abstract

In their pioneering work demonstrating measurement and full correction of the eye’s optical aberrations, Liang, Williams and Miller, [JOSA A 14, 2884 (1997)10.1364/JOSAA.14.0028849379246] showed improvement in visual performance using adaptive optics (AO). Since then, AO visual simulators have been developed to explore the spatial limits to human vision and as platforms to test non-invasively optical corrections for presbyopia, myopia, or corneal irregularities. These applications have allowed new psychophysics bypassing the optics of the eye, ranging from studying the impact of the interactions of monochromatic and chromatic aberrations on vision to neural adaptation. Other applications address new paradigms of lens designs and corrections of ocular errors. The current paper describes a series of AO visual simulators developed in laboratories around the world, key applications, and current trends and challenges. As the field moves into its second quarter century, new available technologies and a solid reception by the clinical community promise a vigorous and expanding use of AO simulation in years to come.

## 1. Introduction

The seminal paper of Liang et al. in 1997 [1] demonstrated for the first time measurement and correction of the ocular high order aberrations (HOA) with Adaptive Optics (AO) and improvement of both the resolution of retinal images and of visual performance. In the latter, the investigators showed improvement in contrast sensitivity at two spatial frequencies when the HOAs of the eye were corrected. This advancement kicked off a new era for exploration of the limits of spatial vision by bypassing the optics of the eye, which up until that time had been confined to the use of small pupils or retinal projection of gratings using an interferometry method [2]. Over the following decade a number of laboratories developed AO systems that allowed psychophysical experiments under corrected aberrations. Teams led by David Williams and Geunyoung Yoon at the University of Rochester [3,4], Pablo Artal at the University of Murcia [5], Chris Dainty at Imperial College/National University of Ireland in Galway [6], Susana Marcos at the Institute of Optics-CSIC [7], David Atchison at the Queensland University of Technology [8], and Jack Werner at the University of California at Davis [9] pioneered several of these first devices. At that time much of the research using AO visual simulators was directed to the understanding of the visual benefits of correcting the aberrations of the eye (on visual acuity, contrast sensitivity, visual tasks such as face recognition), impact of aberration correction on certain functions such as accommodation, adaptation of the visual system to the natural aberrations of the eye, correction and induction of aberrations, and effect of induction of spherical aberrations on, for example, the extension of the ocular depth-of-focus. This research was particularly relevant at a time when laser refractive correction promised the ability of not only compensating the low order refractive error (defocus and astigmatism), but a customized ablation which could minimize the eye’s aberrations [10]. Adaptive optics provided a much-needed foundation for the understanding, if feasible, of the benefits and consequences of the correction of the HOAs of the eye. The early 2000s also saw the development of the first so-called Adaptive Optics Phoropters. Those systems attempted to capitalize on the capabilities of the Hartmann-Shack wavefront sensor to measure low and high order aberrations of the eye, allowing computation of retinal image quality metrics which captured more accurately the eye’s best focus (and therefore refraction). Deformable mirrors were then used as sophisticated programmable trial lenses. The term AO phoropter was first coined by Lawrence Livermore National Lab, which presented a first prototype as part of endeavors of the National Science Foundation Center for Adaptive Optics [11]. The company, Imagine Eyes (Orsay, France) launched the crx1 Adaptive Optics Visual Simulator, which although no longer commercially available is the basis for some of the AO research performed in some labs today [12].

Roorda presented a comprehensive review of the state of the art of Adaptive Optics in Visual Optics in 2011 [13]. There are also practical reviews in the literature that address the practicalities of the technical implementation of AO devices [14–18]. The current review focuses on more recent and current developments and studies. New developments include binocular devices and the incorporation of polychromatic aberrations (measurement and correction) in the image quality assessment. New applications include the study of neural adaptation and perceptual learning in specific group populations (myopes, presbyopes or keratoconus), and very specially, the use of adaptive optics elements (Spatial Light Modulators, in particular) to mimic state-of-the-art or future multifocal designs. Some of these developments have made their way to clinical practice and commercial devices.

In this publication, we have collected current designs of Adaptive Optics visual simulators in different laboratories around the world, selected some recent key applications of those systems in vision research and discussed the authors’ views of current and future improvements of this technology.

## 2. Vobiolab/CVS-Rochester visual optics simulator

### 2.1 Description of the system

**Fig. 1.**
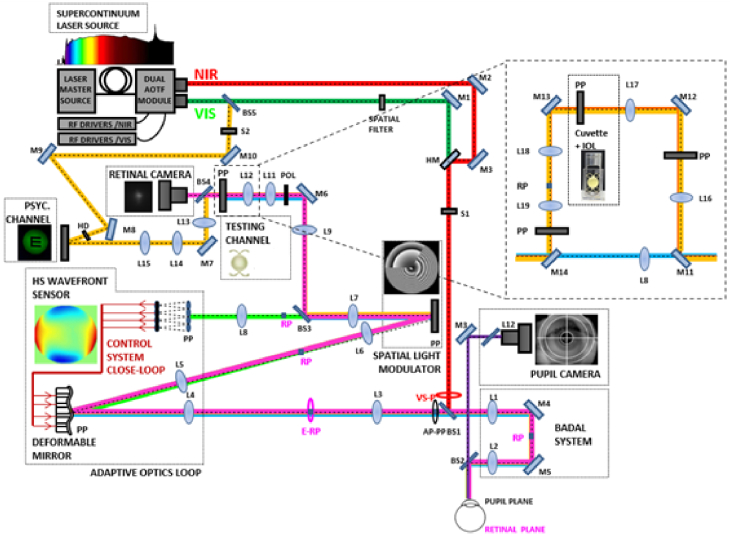
Schematic diagram of the latest version of the VioBio Lab AOII system [8]. Label abbreviations: M stands for Mirrors, L for Lenses, BS for Beam Splitters, HM for hot mirror,, POL polarizer, HD for high-density filter. RP are retinal planes; PP are pupil planes.

The first Adaptive Optics Visual Simulator developed by Marcos et al. was published in 2008 [7], and subsequent versions (VioBio polychromatic AO II) in 2015-2021 [19–24], revised in part in a recent review article [25]. An upgraded version has now started operation at the Marcos Lab in the Center for Visual Science at the University of Rochester [26]. The main feature of this AO Visual Simulator is its multi-channel (allowing manipulation of the wave aberrations with multiple active and passive devices) and its multi-wavelength configuration (allowing monochromatic and polychromatic measurements). The system is formed by the following channels: (1) Illumination channel, provided with a supercontinuum laser source (SCLS, SC400 femtopower 1060 by NKT Photonics, Denmark, in the VioBioLab set-up, and L-CO-28-0082-000, by Leukos, France in the CVS set-up), coupled with a dual acousto-optic tunable filter (AOTF) module to automatically select the wavelengths in the visible (450-700 nm) or Near infrared (700-1100 nm) allowing wavefront sensing, and retinal aerial imaging at multiple wavelength, as well as psychophysical experiments in selected visible wavelengths [20,27]; (2) The Hartmann-Shack (HS) wavefront sensor (microlens array 40 × 32, HASO 32 OEM by Imagine Eyes, France); (3) The Deformable-Mirror (DM, 52 actuators, MIRAO) to correct the subject’s aberrations and/or mimic smooth refractive optical correction elements; (4) A Spatial-Light Modulator (SLM) channel, provided with a reflective phase-only LCoS-SLM (SLM; VIS; Resolution: 1920 × 1080; Pixel pitch: 8.0 µm; by Holoeye, Germany) to generate static segmented or diffractive lens designs [21]; (5) A physical testing channel for inserting phase-plates, and in a recent configuration, provided with a cuvette that allows inserting intraocular lenses of range of powers to be physically projected onto the eye’s pupil [24]; (6) A double-pass retinal imaging channel allowing capturing retinal images of a point source, consisting of a spatial-filtering module, a CCD camera (Retiga 1300) and a collimating lens [20]; (7) A visual stimulus channel for fixation and psychophysical measurements, consisting of a Digital Micro-Mirror Device (DMD by Texas Instruments), placed in a conjugate retinal plane, subtending a 1.62 deg field. The DMD is monochromatically illuminated with light coming from the SCLS through a holographic diffuser (HD) that breaks the coherence of the laser and provides uniform illumination of the stimulus. In a recent version of the system a red/blue concentric square slide was inserted for (in combination with a cross-target projected in the DMD) allows vernier alignment for transverse chromatic aberration measurements [23]; (8) A pupil monitoring channel (LED illuminator and a CCD camera, conjugate to the eye’s pupil); (9) A spherical correction/accommodation induction module (a Badal Optometer in the VioBio Lab system, and an Optotunable lens (Optotune, Switzerland) and offset lens in the CVS system. Figure 1 shows a schematic diagram of the AO Visual Simulator.

### 2.2 Key application

There are two major areas where the system has been applied: (1) Understanding the limits to spatial vision imposed by the ocular optics: effects of monochromatic and chromatic aberrations on visual perception and visual performance [28–32]; adaptation of the visual system to native or imposed ocular aberrations [33–40]; (2) non-invasive testing of vision through simulated intraocular or contact lens designs [22,41–46].

**Fig. 2.**
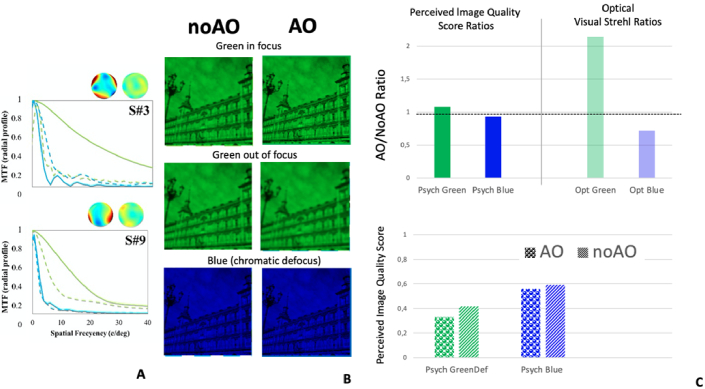
A. MTF radial profiles calculated from the measured wave aberrations for two patients (maps on top of each graph with natural HOAs and AO-corrected HOAs). Solid lines stand for AO-corrected HOAs, and dashed lines for natural HOAs. Green lines are for 555 nm at best focus. Blue lines are for 480 nm (including chromatic defocus). B. Illustration of images presented in the psychophysical score experiment through natural and AO corrected aberrations. Green images are presented in focus and out of focus (defocus equivalent to green-blue chromatic blur). Blue images are naturally defocused, as the best focus is set for green. C. Perceived Image quality score and Optical Visual Strehl Ratios AO/NoAO (upper graph), for green (in focus) and blue (defocused by chromatic defocus). Correcting HOAs improves the optics by a factor 2, and very moderately perceived image quality in green; and degrades the optics and perceived image quality (upper graph) in blue; Perceived image quality score on defocused green images (equivalent chromatic defocus) and in blue (naturally defocused) (bottom graph). Blue images are consistently scored higher than defocused green images) for both AO and noAO. Data are average of 10 patients, and for 6-mm pupils, adapted from [33], where individual data are presented.

The two examples below are representative for each of these two areas:

*Effect of monochromatic and chromatic aberrations on visual quality.* Monochromatic, chromatic aberrations and their interactions all affect retinal image quality, and subsequently vision. Earlier work suggested that monochromatic aberrations protected the eye from the impact of longitudinal chromatic aberrations due to favorable optical interactions [47], an effect which was recently confirmed to be dependent on the magnitude of the HOAs (examples in two subjects are shown in Fig. 2(A)) [33]. The AO Visual Simulator allowed us to correct the monochromatic aberrations of the eye with the deformable mirror (DM) and test the perceived image quality of natural images in green and blue light [33] (Fig. 2(B)). Figure 2(C) shows the optical (Visual Strehl ratios) and visual benefit (visual score ratios) of correcting monochromatic aberrations in monochromatic green stimuli, and in monochromatic blue stimuli (subject to chromatic aberration). In green light, the benefit of correcting the ocular optics in perceived quality is >1, but significantly lower than the optical predictions, likely as a result of neural adaptation to the natural aberrations. In blue light (in the presence of optical blur produced by the longitudinal chromatic aberration), correcting monochromatic aberrations reduces both the optical quality and perceived visual quality, likely as a result of the loss of favorable interactions between chromatic and monochromatic aberrations (ratio <1). Furthermore, the perceived image quality of defocused images in green light (by an amount equivalent to the chromatic defocus, −0.87 D) is consistently lower than those of naturally defocused (by chromatic blur) images in blue (Fig. 2(C)) [33]. This effect occurs both under natural and corrected high order aberrations, and may reflect a contingent adaptation to both defocus and blue light.

**Fig. 3.**
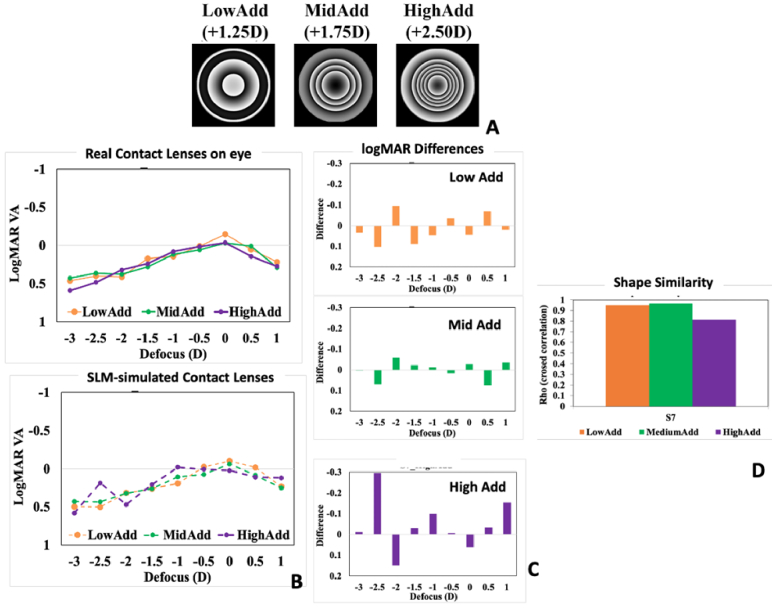
SLM-based simulations of commercial center distance low, medium and high add contact lenses for presbyopia. A. Phase-wrapped phase maps of the three contact lens designs, as mapped on the SLM. B. Through Focus Visual Acuity with the real multifocal contact lenses on the eye (Top) and the SLM-simulated Contact lenses (Bottom). C. Difference between Simulation and Real CL (logMAR) as a function of defocus; (D) Shape similarity metric (correlation) between SLM and Real MCL on the eye. The figure illustrates an example of an individual eye (S7) from the Vedhakrishnan et al. study [44].

*Visual performance with simulated multifocal lenses.* Multifocal lenses are an increasingly used alternative for presbyopia management, providing foci at near and far or extending the depth-of-focus to provide intermediate and near vision functionality in patients that have lost accommodation [48]. More recently certain contact lens designs have also been used in myopia control [49]. The AO Visual Simulators are well suited to map different lens profiles onto the subject’s eye and compare their effects on visual performance, prior to implanting them (intraocular lenses, IOLs) or placing them in/on the patient’s eye (contact lenses, CLs). We have used the SLM in the AO Visual Simulator to represent the power profile of multifocal CLs (center near; low, medium and high adds, Fig. 3(A)) and study the through focus visual acuity (VA) with different designs on the same eye (Fig. 3(B)) [49]. An intra-subject comparison of the performance of the simulated lenses and real CLs on the eye showed a high degrees of similarity, both in absolute values (0.04 ± 0.01, 0.03 ± 0.01, and 0.03 ± 0.04, logMAR VA differences for LowAdd, MidAdd and HighAdd, averaged across 7 subjects, and 4-D through-focus, Fig. 3(C)), and shape similarity of the through-focus curves (rho = 0.889 ± 0.03, rho = 0.825 ± 0.05 and rho = 0.651 ± 0.08, for LowAdd, MidAdd and HighAdd lenses respectively, on average, Fig. 3(D)).

### 2.3 Current and future improvements

The VioBio/CVS AO Visual Simulator has grown to accommodate multiple channels, allowing measurements of monochromatic, longitudinal and transverse chromatic aberrations, closed-loop correction of HOAs, simulation of refractive, segmented and diffractive optical corrections, refraction correction/accommodation stimulation, double-pass retinal image monitoring, and visual psychophysics (visual function, perceived visual quality, neural adaptation). The system will incorporate also chromatic aberration correction through active components [50,51]. A drawback of SLM-based simulators is the wavelength-dependence of the programmed phase. Also, while optical bench systems are versatile, their specifications and footprint are less suited to be used in a clinical environment, and they are generally not adapted to see the real world. The angular extent of the display is typically around 2-deg, and the presented stimuli are confined to 2-D images projected internally in the system. Furthermore, most on-bench AO systems are monocular, although some laboratories have presented binocular systems [52–55]. In addition to binocularity, recent instruments have also overcome some of these drawbacks, and deliver a binocular, see-through experience of the real world through manipulated optics. In particular, the Simultaneous Vision Simulator (commercialized by 2EyesVision under SimVisGekko) uses the principle of temporal multiplexing with an optotunable lens to represent multifocal and extended depth of focus lenses [42,45,56–58]. The system is binocular, head-mounted, open field, and has a 20 deg field of view. Future systems of interest in research and the clinic should be binocular and see-through, incorporate a Hartman-Shack wavefront sensor, and (among other possible transmissive adaptive optics elements) deformable lenses to induce/correct HOAs.

## 3. Murcia University (LOUM) adaptive optics visual simulators

### 3.1 Description of the system

The **“**Laboratorio de Optica de la Universidad de Murcia” (LOUM) is one of the world pioneers in the development of adaptive optics technologies for the eye. As early as in 1998, we published the first article demonstrating the static correction of ocular HOAs using a pixelated phase modulator in transmission [59]. A few years later, we also demonstrated one of the first closed-loop aberration correction dynamically with a membrane deformable mirror [60]. Although the main application in the early days of AO for the eye was to improve the resolution and quality of retinal fundus images, another important application of AO was devised: visual simulation. We realized that not only HOAs could be corrected, but also any desired aberration pattern could be added to the eye in a controlled manner. Through an additional optical path, visual stimuli were projected to the subject’s eye to perform visual testing for a variety of optical conditions. This is the basis of the concept of the adaptive optics vision simulator. We introduced first the idea and indeed coined the name of AO visual simulators [5].

**Fig. 4.**
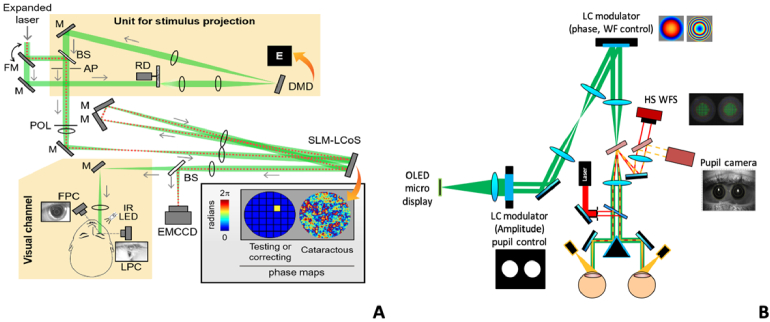
A. Schematic of the Murcia AO visual simulators: A. AO system with amplitude and phase control for the simultaneous generation and compensation of the effects of intraocular scattering with WS. B. Binocular AO visual simulator. Label abbreviations: FM stands for flip mirror, M for mirror, RD for rotating diffuser, BS for beam splitter, CA for circular aperture, POL, linear polarizer, IR LED for infrared light-emitting diode, LC for liquid crystal, SLM for Spatial Light Modulator, EMCCD for electro-multiplying charged-coupled device, FPC and LPC for frontal and lateral pupil cameras. The red dotted line depicts the path of the beam during the feedback based WS correction

The LOUM Adaptive Optics Visual Simulator (AOVS) consists of a wavefront sensor (WS) to measure the eye’s aberrations and a correcting device to modify the eye’s optics. The Hartmann-Shack wavefront sensor [61] operates in infrared light to measure the eye's aberrations and residual defocus in real time. The correcting/manipulating device is placed in the system conjugate both with the subject's pupil plane and the wavefront sensor, by using appropriate sets of lenses in a telescope configuration. Subjects view a stimulus (letters or any visual scene) produced either by a pico-projector or a micro-display. Different types of correcting devices, including phase modulators [62,63] and deformable mirrors have been used separately and also in combination [64]. The first version of a binocular version of the instrument was proposed [65] using one single sensor and corrector for both eyes, without the need of replicating a monocular system. More recently the binocular AOVS was further improved adding full control of the pupil amplitude and phase [66]. Figure 4(A) shows a schematic diagram of the binocular version of the AO visual simulator. New capabilities of the AOVS, include extended defocus range of operation with the addition of tunable lenses [67] and we have contributed to the transition to a commercial clinical apparatus developed by Voptica SL, the VAO [68]. Beyond the correction of aberrations, intraocular scatter affects the eye’s image quality, with special severity in cataracts. We have recently proposed the use of wavefront shaping (WS) as a type of high-resolution AOVS for correction of both aberrations and scatter. We were able to improve the eyés PSF through simulated cataracts of different clinical grades [69]. Figure 4(B) shows the schematic diagram of the instrument WS-based AOVS.

### 3.2 Key application

Since with AOVS, not only defocus and astigmatism, but all optical aberrations can be corrected and/or induced, this allows optimizing the optical correction for different visual tasks and conditions. In invasive procedures, such as laser refractive surgery, before a definitive ablation of the cornea is performed, the optical profile to be induced could be optimized for each patient. One of our main objectives with the use of the AO visual simulators was pre-testing of different visual corrections such as intraocular lenses [70] or refractive surgery [71] before any permanent treatment is performed. This would increase safety and improve the visual outcomes. We have also extensively used in the lab the binocular AOVS for design and optimization of contact and intraocular lenses.

In addition to this practical application, we have been also interested in the use of AOVS for a better understanding of the relationship between the eye’s optics and vision. One early milestone was the discovery that the visual system is actually adapted to the particular eye’s aberrations [72]. This research was extensively followed and confirmed by other groups later.

On the other hand, we used a version of the AOVS to explain the possible causes of night myopia [73] and the combined effect of the spherical and chromatic aberrations on the quality of vision [74] among other studies. The binocular AOVS allowed us to explore the impact of aberrations on stereo acuity [53] and the limits of several presbyopic correcting approaches on stereo vision [75].

### 3.3 Current and future improvements

The improvement of the AOVS is a continuous process. We are working on the incorporation of fast and reliable pupil tracking techniques [76] into the systems and faster processing using GPUs [77]. Our main current line of research is the integration and miniaturization of the AOVS to be converted into wearable devices. Although this is posing many technical challenges, we are making good progress in the development of wearable W-AOVS that can operate under natural viewing conditions. These new types of systems would open new options for diagnosis and visual outcomes optimization in ophthalmology.

## 4. Atchison lab visual simulator

### 4.1 Description of the system

**Fig. 5.**
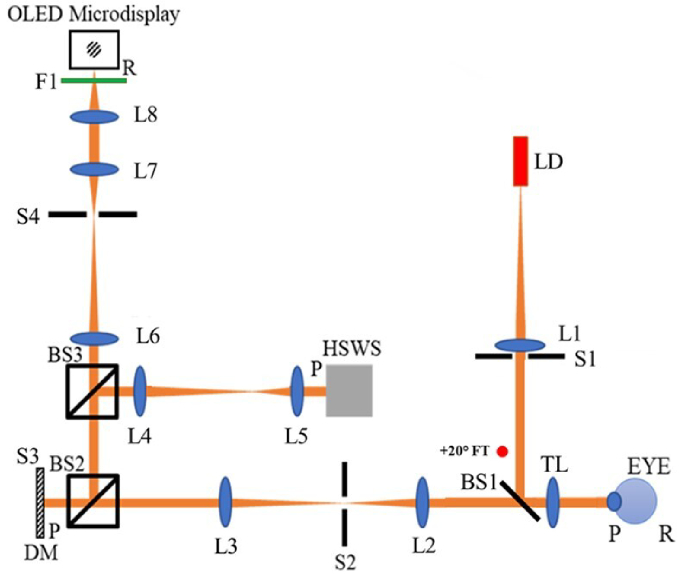
Schematic diagram of the adaptive optics setup used to correct on-axis and peripheral aberration. LD - Red laser diode (λ = 635 nm), L1 to 8 - achromatic lenses, S1 to 4 - apertures (S1 - Laser diode luminance controller, S2 - corneal reflection controller, S3 - stop, S4 - field size controller), M1 - plane mirror, BS1 to 3 - beam splitter, TL - + 2D or –2D trial lens to induce defocus, FT - peripheral fixation LED targets (+20°), DM - ALPAO DM69-15 deformable mirror, HSWS - Hartmann-Shack wavefront sensor, F1 - Monochromatic filter (λ = 532 nm), P – pupil/pupil conjugate plane, R – retina/retinal conjugate plane.

Figure 5 shows the Atchison Lab Visual Simulator optical setup, which is comprised of three main arms. The illumination system arm contains a 635 nm laser diode placed at the focal point of lens L1 and a circular aperture S1 controlling the beam diameter, with beam splitter BS1 directing the collimated laser beam to enter the eye.

The wavefront sensor arm has three beam splitters BS1 to BS3 and four lenses L2 to L5 to ensure that the deformable mirror (DM) and the lenslet array of the Hartman-Shack wavefront sensor (HSWS) are conjugated to the entrance pupil of the eye. HOAs are corrected over the entire DM surface whose aperture (S3) is 10.5 mm diameter. This acts as a 7 mm pupil stop because of 1.5x magnification between the DM and the eye.

The stimulus arm has an OLED micro-display to display Gabor gratings of 100% central contrast. Filter F1 of wavelength 532 nm (10 nm full width at half maximum), in front of the display restricts chromatic aberration. Light is reflected at BS2 onto the DM. Lenses L6 to L8 conjugate the display with the retina. Aperture S4 controls the field size of the target on the OLED display.

### 4.2 Key application

The theme of the Atchison Lab work has been to evaluate the appropriateness of testing methods to measure peripheral optics and visual performance. for the adaptive optics component of this work, we explored the effect of optical blur on the contrast sensitivity function in the peripheral field, with the hypothesis that we would be able to identify local depressions (notches) that have been noted in central vision [78]. For this, adaptive optics was applied to isolate the influence of defocus from other aberrations.

Furthermore, after having noted that the use of interferometers to “bypass” the ocular optics gives higher visual acuities than screen-based studies using Gabor functions, we investigated the effect of the degree of Gabor attenuation on the difference [79]. As aberrations will affect acuity for the screen-based testing but not for the interferometer, adaptive optics was used to minimize the aberrations for the former to get the closest possible matches.

**Fig. 6.**
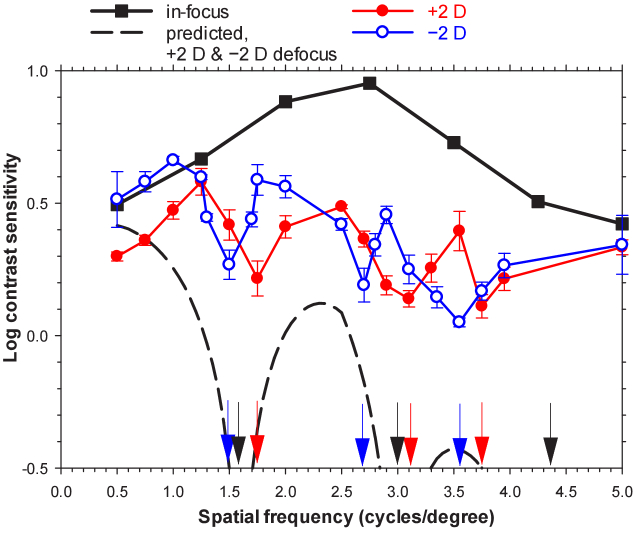
Measured and predicted peripheral contrast sensitivity functions for one participant with vertical grating orientation: in-focus and with +2 D and −2D blur induced by trial lenses. Error bars represent the standard deviations for three repetitions. Error bars are not visible when the standard deviation is less than 0.05 log contrast sensitivity. Log contrast sensitivity is shown down to −0.5 to better show the pattern of predicted values, although the shaded region below 0 (where there is 100% contrast) cannot occur physically. Arrows indicate measured +2 D (red), measured −2D (blue), and predicted (black) spatial frequencies of notches. Adapted from Jaisankar et al. [78].

Figure 6 shows results for the work investigating the effect of optical blur on the contrast detection sensitivity function. These are for one participant, 20° nasal visual field position, vertically oriented Gabor gratings and for three defocus levels (0 D, + 2 D, and −2 D). The results are similar for both vertically and horizontally orientated gratings and for two other participants. Notches in the monochromatic defocused Contrast Sensitivity Functions (CSF) occur at 1.5-1.7, 2.7-3.1 and 4.4-4.5 cycles/degree, close to the predicted spatial frequencies. Notch depths (0.2 to 0.5 log unit) are smaller than predicted. Unless recognised, such notches may contribute to noise in through-focus detection measurements of peripheral visual performance.

### 4.3 Current and future improvements

The setup can be readily improved by providing a stop to give different effective pupil sizes; the current 7 mm is overlarge. The lenses to induce blur can be replaced by a trombone system or active liquid lenses. Experiments required several hours per participant, and efforts should be put into refinement of adaptive psychophysical procedures. Furthermore, an interferometry channel can be added to do comparability measurements.

## 5. University of Bradford/Manchester visual simulator

### 5.1 Description of the system

**Fig. 7.**
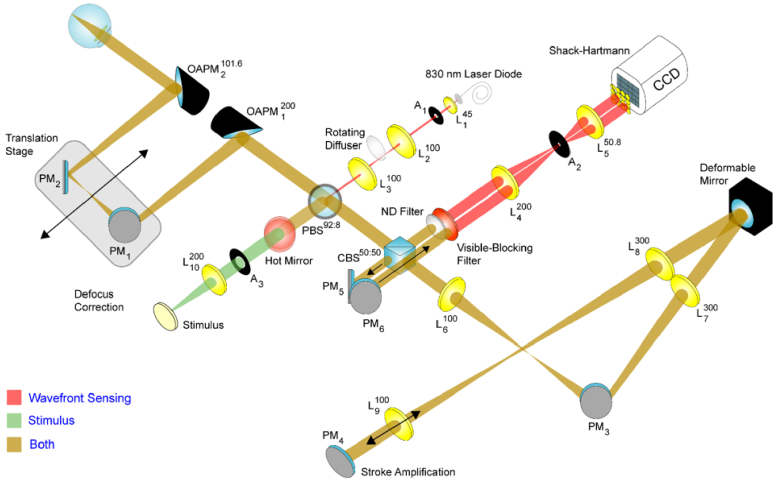
Schematic diagram of the dual wavefront sensing channel monocular adaptive optics system. L, lens, (focal length in millimeters); PM, plane mirror; OAPM, off-axis parabolic mirror; A, aperture; PBS and CBS, pellicle and cube beamsplitter respectively (transmission: reflection).

The Bradford/Manchester dual wavefront sensing channel monocular adaptive optics system is shown in Fig. 7 [80]. The system is mounted on a research-grade breadboard that is 600 × 900 mm in size (Newport Ltd, UK).

*Wavefront sensing eye illumination path**:*** The wavefront sensing light source is an 830 nm fiber-coupled laser diode (Access Pacific, UK). After the light exiting the fiber tip is collimated by lens *L_1_*_,_ it is focused by *L_2_* onto a rotating diffuser to reduce speckle [81]. All lenses in the system are spherical achromatic doublets, aside from *L_1_* which is a molded glass aspherical lens. Once re-collimated by lens *L_3_*, the light enters the main path of the system via a pellicle beamsplitter, *PBS*. The light is then focused by an off-axis parabolic mirror, *OAPM_1_*, and passes through to plane mirrors, *PM_1_* and *PM_2_*, that are mounted on a translation stage. Movement of these plane mirrors is used to correct for myopia and hyperopia, and to set the accommodative demand. Following reflection from these plane mirrors, the light is reflected into the eye via *OAPM_2_*. OAPMs are used in the illumination path to mitigate back-reflections that would otherwise be directed towards the Shack-Hartmann wavefront sensor. In order to prevent back-reflections from the eye from reaching the sensor, the beam enters the eye off-axis, thus allowing this light to be blocked by aperture *A_2_*. The beam entering the eye is 1 mm in diameter, as set by aperture *A_1_*, and has a power of 120 µW.

*Wavefront sensing paths**:*** The Shack-Hartmann wavefront sensor measures the aberrations at 20 Hz. The sensor consists of a CCD camera (Retiga Exi, QImaging, Canada) and a 7 mm focal length lenslet array with a pitch of 200 µm. The magnification changes through the system result in the pupil being sampled at 0.4 mm intervals. A unique feature of the system is that it contains two wavefront sensing paths. The wavefront sensing light returning from the eye is separated into two paths using a cube beamsplitter, *CBS*. Fifty percent of the light is reflected directly toward the Shack-Hartmann sensor. It impinges upon the lenslet array after passing through lenses *L_4_* and *L_5_*. This forms the first sensing path. As this light bypasses the deformable mirror, it is possible to obtain a direct measurement of the eye’s aberrations rather than inferring them from measurements contaminated by the response of the deformable mirror. From these measurements, the accommodation response of the eye can also be directly determined.

The light transmitted by *CBS* passes via lenses *L_6_* and *L_7_* onto a 37-actuator deformable mirror (Flexible Optical BV, The Netherlands). The mirror is 30 mm in diameter with a stroke of 8 µm. In order to effectively double its stroke, the light strikes the deformable mirror twice. This is achieved via lenses *L_8_* and *L_9_* and a plane mirror *PM_4_*. On its return path, the light reflected from *CBS* is directed towards the Shack-Hartmann sensor using *PM_5_* and *PM_6_*. This forms the second sensing path and is used to control the deformable mirror in a closed-loop. Note that both sensing paths use the same Shack-Hartmann sensor to reduce cost and complexity. This necessitates the use of a neutral density (ND) filter in the first sensing path to balance the brightness in the two paths. The deformable mirror is also used to introduce rapid step changes in the accommodative demand.

*Stimulus path:* The stimulus consists of a white light source illuminating a Maltese cross on a black background. A 550 nm filter is used to render the light monochromatic and results in a luminance at the retina of 6.7 cd/m^2^. The stimulus enters the main beam path via *PBS* and subtends 1° at the retina. A 45° hot mirror is used to direct the infrared light from the wavefront sensing light source out of the stimulus path. This is to prevent infrared light from being reflected from the stimulus and entering the Shack-Hartmann path. Visible light from the stimulus is prevented from reaching the Shack-Hartmann sensor using a visible-blocking filter.

### 5.2 Key application

The goal of the system is to investigate the role of ocular monochromatic aberrations in accommodation. Aberrations present in a typical eye can result in an image appearing different depending on the sign of the defocus present [82]. Consequently, aberrations have the potential to guide the accommodation system.

**Fig. 8.**
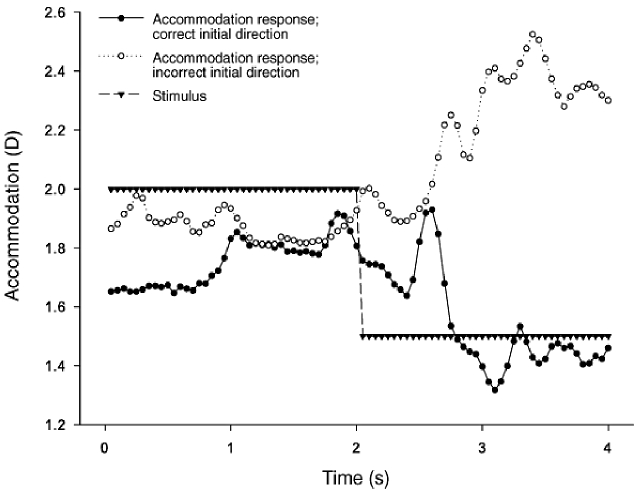
Accommodation responses to a step change in stimulus demand for a single subject when all aberrations from second order (excluding defocus) up to and including sixth radial order are inverted following the stimulus step change [84]. An example of a correct and incorrect accommodative response is shown.

*Accommodation response to a dynamic stimulus**:*** We have explored the effect of dynamic correction of aberrations on +/- 0.5 D steps in accommodative demand [83]. Second order Zernike aberrations (excluding defocus) and up to and including sixth radial order Zernike aberrations were dynamically corrected at various time points: after the stimulus step change, before the stimulus step change, during the accommodative response latency period, and throughout the experimental run. We found that continued correction of ocular aberrations after the step change significantly changed the gain of the response. In another study we again used step changes in accommodative demand, but this time investigated the effect of dynamically inverting the aberrations of the subject after the step change in accommodative demand. In this case, we found that the accommodative response would initially be in the wrong direction during some trials, as shown in Fig. 8 [84]. We have also investigated the accommodative response to a stimulus fluctuating sinusoidally between 1.5 D and 2.5 D at a frequency of 0.2 Hz [85]. That frequency was chosen as it is within the low frequency region of accommodation micro-fluctuations, which are considered to be under neurological control and an aid to the accommodation system [86]. For one of our subjects, dynamic correction of aberrations reduced the gain of the accommodative response, but for the others, there was no effect.

*Accommodation response to a static stimulus:* The system has been used to determine the effect of dynamic correction of aberrations on the micro-fluctuations in accommodation when viewing a static stimulus [87,88]. In this study, manipulation of aberrations other than variations in focus did not generally affect the micro-fluctuations properties.

### 5.3 Current and future improvements

Studies investigating the effect of monochromatic aberrations on the accommodation system [89], will benefit from natural viewing conditions.

A limitation of the instrument presented here is that it is only able to manipulate the aberrations monoculary. When viewing the world around us, not only do both eyes accommodate, but they also change their convergence and pupil size. This triad of changes is inter-linked. While a small number of binocular adaptive optics systems do exist, see for example [90,54], they are not capable of dynamically manipulating convergence. Currently, we are developing a binocular adaptive optics system with this capability. The system uses deformable mirrors with an increased number of actuators and strokes in comparison to the monocular system presented here (2 x MirAO-52, Imagine Eyes, France). Galvanometer-controlled mirrors conjugate to the center of rotation of each eye are used to control convergence. The CCD camera used for wavefront sensing measures the aberrations of both eyes, while simultaneously capturing an image of both pupils to measure convergence and pupil size.

## 6. LuMIn, ENS Paris-Saclay - crx1 adaptive optics visual simulator

### 6.1 Description of the system

**Fig. 9.**
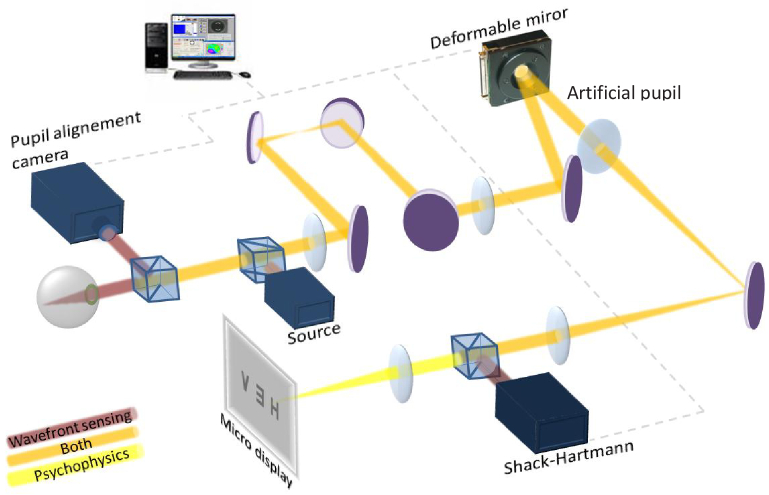
Schematic diagram of the crx1 device.

We use a deformable mirror (Mirao, Imagine Eyes, France) in closed-loop with a wavefront sensor (HASO CSO, Imagine Eyes) either to dynamically correct the subject’s wavefront aberration and/or to induce various levels of monochromatic aberrations (Fig. 9). The system optically conjugates the subject’s exit pupil plane with the correcting device, the wavefront sensor and an artificial pupil. The Shack–Hartmann wavefront sensor has a square array of 1024 lenslets. The wave-aberration measurements are made at 850 nm.

The wavefront corrective device is a deformable mirror using 52 independent magnetic actuators. The control of the deformable mirror surface is accomplished by a commercially available program (HASO CSOTM, Imagine Eyes) which reshapes the deformable mirror from its normally flat surface to a shape that corrects the aberrations up to the 6th order (25 Zernike coefficients) [91]). The micro-display (eMagin, Rev2 SVGA + White Oled Microdisplay) subtends a visual angle of 114 × 86 arcmin with a resolution of 800 × 600 pixels (pixel size = 0.143 arcmin). The pupil position and size are monitored using a CCD camera. The pupil center is aligned with the optical axis of the set-up. The subject’s pupil is not artificially dilated since the experiments are performed in dim surrounding illumination providing a diameter higher than 6 mm. The mirror changes its shape for any variation of the aberration pattern of the subject, so the accommodative response to a stimulus is also compensated by the mirror.

### 6.2 Key application

A typical use of adaptive optics is to statically [91–93] or dynamically [94–98] compensate for monochromatic aberrations of the observers. As an example, we measured the visual benefit of correcting HOAs on the contrast sensitivity and visual acuity [99], and determined whether the correction of HOA would be detectable by “normal” subjects and whether a full correction is necessary or better than a partial correction of the main HOA [100].

AO has also allowed us to investigate whether subjects with high levels of HOAs (e.g., keratoconic eyes) are adapted to their usual aberrations. We determined whether “normal” eyes viewing through the aberration pattern of keratoconic eyes were able to achieve a similar visual performance to that of keratoconic eyes [99].

We also used AO to explore the effect of various levels of aberrations on subjective vision by scoring images or measuring visual acuity or contrast sensitivity [93,100–103], and to examine the accuracy of image quality metrics in predicting visual performance on these tasks [96,99,100]. Therefore, we were able to compare real optical blur (i.e., induced by a deformable minor) to simulated blur [94,100,102,103] or image quality metric prediction [94,96,98].

*Comparison between real optic and simulated blur**.*** Clinical studies of new lens designs are time consuming. An alternative to the clinical testing of new designs (e.g., optical designs aiming to compensate for presbyopia) could be the numerical simulation of their on-eye performance, although the accuracy of the numerical simulation should be tested. We conducted various experiments in order to test the ability of a numerical eye model to predict the effect of monochromatic aberrations on visual performances and subjective image quality [94,96,100,102,103]. The term numerical simulation encompasses both calculations of image quality metrics (IQM) based on the MTF, OTF or PSF and the convolution of the PSF with an original (i.e., un-aberrated) image. While numerical image quality metrics leave no place for subjectivity, simulated images judged by real observers seem closer to clinical testing where the subjective patient responses are required.

We used our AO system (crx1, Imagine Eyes) to measure visual acuity, contrast sensitivity, depth-of-focus (i.e., interval of vision for which the target was still perceived acceptable) and image quality score in the presence of defocus, astigmatism, coma, spherical aberration (SA4) and secondary spherical aberration (SA6). We used the deformable mirror to dynamically compensate for the observer’s aberrations and to simultaneously induce the desired aberration. The closed-loop system works at 1 Hz that comprised of the double-pass of light through the eye, so that the total (eye-device) aberrations encountered along the line of sight was continuously minimized. These dynamically adjusting wavefronts enable the compensation of small eye decentration and aberration variations due to the tear film or accommodation. During the experiments, the typical average variance of the residual aberration is around 0.10 µm on a 6 mm pupil size. The subject views the simulated or original images on a micro-display (typically a white screen of 100 cd/m^2^) through an artificial pupil conjugated to the observer’s pupil.

In general, there was a good correlation between contrast sensitivity using sinusoidal gratings and IQM. This is in agreement with results by de Gracia et al. [104], using the Atchison Lab Visual Simulator, who showed similar trends for optical and visual improvement in the CSF as a function of spatial frequency, and across orientations when aberrations were corrected. However, the visual improvement was much more modest than that predicted by the optics. On the other hand, the correspondence between predicted and optically simulated visual acuity and depth-of-focus was poor [93,98], suggesting a mismatch between the simulated and actual blur on the retinal image or a larger neural component for these tasks.

**Fig. 10.**
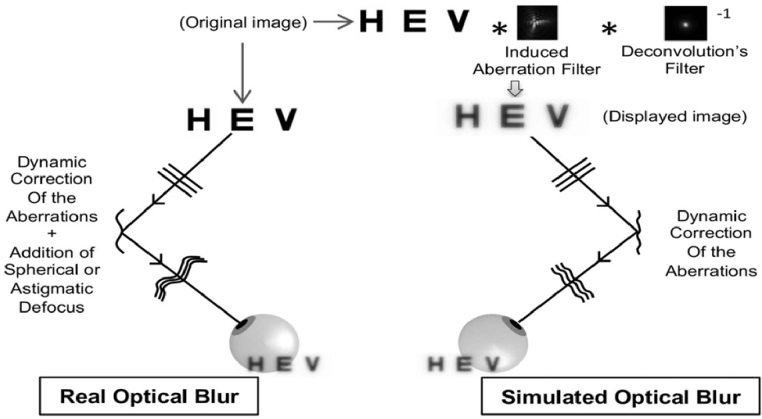
Schematic diagram of the two methods used to compare real and simulated optical blur.

Computational convolutions have often been used to assess the impact of HOAs on the retinal image quality. With the deformable mirror of an AO Visual Simulator it is either possible to induce aberrations and dynamically correct the subject’s aberrations while the subject views an original image (mirror-controlled condition or real optical blur) or to only dynamically correct the subject’s aberrations while the subject views a simulated image (object-controlled condition or simulated optical blur) (Fig. 10) [93,94,96,103].

Although from optical principles subjective depth-of-focus [11] or image quality score [101–103] using convolved images through corrected optics or real aberrations should match [103], Vincent et al. [103] observed a difference of about 20 and 35% between simulated and real optical blur, respectively, in the presence of spherical and astigmatism blur. Aissati et al. [105] demonstrated that, when keeping all other conditions similar in both experiments, the discrepancies found using polychromatic targets further reduced when the experiment was conducted in monochromatic light, as monochromatic and chromatic aberrations interact differently in both conditions.

### 6.3 Current and future improvements

The closed-loop system operates at 1 Hz rate, which is too slow to correct the fluctuations of accommodation. A faster closed-loop (>5 Hz) correction of the eye’s aberrations would improve the quality of the comparison between real optical and simulated blur, it will also permit to study the contribution of the micro-fluctuations of accommodation to the accommodation process, and shed light into their potential role on the eye’s focusing control

## 7. KTH adaptive optics visual simulator

### 7.1 Description of the system

**Fig. 11.**
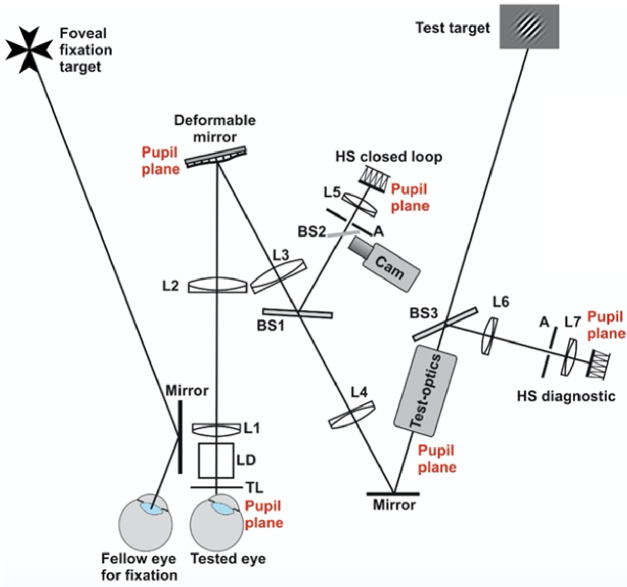
Schematic diagram of the optical set-up of the KTH Adaptive Optics Visual Simulator. TL Trial lens holder, LD Laser Diode 830 nm; L1-7 achromatic lenses; HS Hartmann-Shack sensor; BS1-3 Beamsplitters (1 and 3 are hot mirrors; 2 is a pellicle); A Aperture; Cam Pupil camera.

The Adaptive Optics Visual Simulator of the Visual Optics group at KTH is specially designed for peripheral measurements, but can also be used foveally. It consists of a deformable membrane mirror in closed-loop with a Hartmann-Shack wavefront sensor (Mirao52e and HASO 4 from Imagine Eyes). The lay-out of the optical set-up, which is shown in Fig. 11, also includes an additional wavefront sensor for diagnostic purposes (under development, see “Current and Future Improvements” for more information). The achromatic lenses pairwise form afocal systems (L1 + L2, L3 + L5, L3 + L4, L6 + L7) to conjugate the pupil of the subject to the deformable mirror, the wavefront sensors, and any additional test-optics. Lens L1 acts as a Badal lens and the total angular magnification through L1 to L4 is +1. The wavefront measuremfnts are done in near-infrared, but for defocus calibration visible light from gray-scale test targets shown on a monitor is used. Hence, the calibration compensates for both longitudinal chromatic aberrations and the distance to the test targets. A head-chin rest is used for stabilizing the subject. Additional infrared illumination and a pupil camera aid in positioning the subject correctly. For peripheral measurements, the fellow eye is most often used for fixation with an external foveal fixation target as shown in Fig. 11.

The key features of the KTH Visual Simulator are: (1) the design for peripheral vision evaluation with large stroke of the deformable mirror and psychophysical procedures developed in-lab; (2) the stable continuous closed loop, allowing for optimum aberration correction throughout the psychophysical session; (3) the universal defocus calibration that eliminates the need for any individual defocus adjustments (by foveal through-focus low contrast visual acuity measurements on experienced subjects with cycloplegia and aberration correction); (4) the additional wavefront sensor for diagnostic purposes.

### 7.2 Key application

**Fig. 12.**
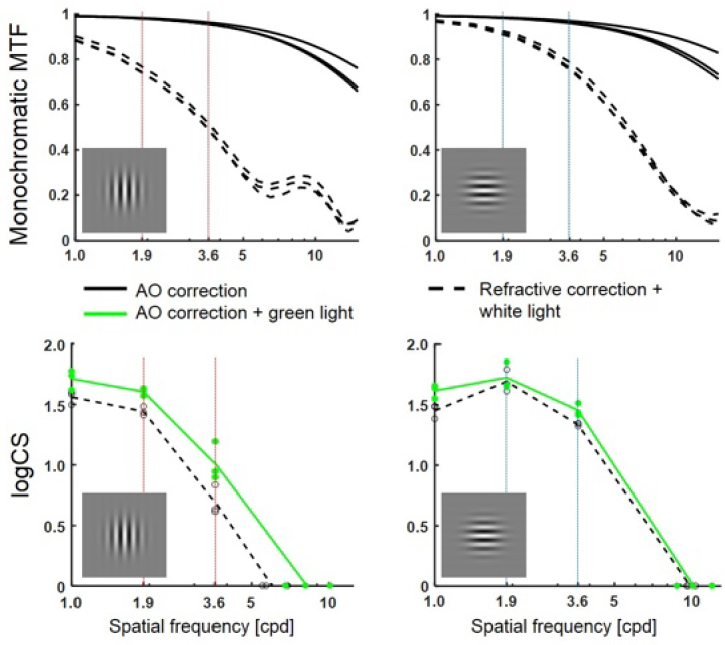
The monochromatic modulation transfer function (MTF) and contrast sensitivity (CS) with adaptive optics (AO) versus refractive correction for one subject in the 20° nasal visual field. The left and right columns show the results for gratings orientated perpendicularly to and parallel to the field angle, respectively. The solid lines represent the best image quality in green light, whereas the dashed lines are with monochromatic and chromatic aberrations still affecting the retinal image. Data from Venkataraman et al. [111].

The KTH Visual Simulator has been in operation since 2011 (first described in Rosén et al. in 2012 [106]). We mainly use the system to investigate how peripheral vision, typically resolution and detection acuity and contrast sensitivity, is affected by optical errors and stimulus properties. An example of how the peripheral modulation transfer function and the contrast sensitivity are affected by monochromatic and chromatic aberrations, and by the orientation of the stimulus, is shown in Fig. 12. The specific aims of the research projects are (1) to improve optical correction designs by better understanding the optical limitations of peripheral vision [107–112], (2) to understand and optimize the optical treatment effect of myopia control interventions [113], and (3) to improve the remaining peripheral vision for people with central visual field loss through optical correction [114].

### 7.3 Current and future Improvements

The KTH Visual Simulator has recently been updated with a new deformable mirror and a new wavefront sensor to ensure stable operation and large stroke of the mirror. For the future we see a need to test commercial optical corrections at the same time as the individual aberrations of the eye itself are controlled. Therefore, there is space on the optical table to mount additional test-optics in the pupil plane after lens L4. Such test-optics may for example be multifocal lenses for myopia control or presbyopia compensation. To be able to monitor the effect that the test-optics have on the retinal image quality during the vision evaluation, we are currently incorporating a separate diagnostic unit (the HS diagnostic in Fig. 11). This diagnostic unit can be a wavefront sensor, as shown in Fig. 11, or a camera to record a double-pass image of the point-spread function of the eye.

Another future improvement of the system is to update the monitor that shows the test targets for the vision evaluation. We are currently using an analogue cathode-ray-tube monitor (Nokia 446Xpro) driven by a Linux PC with a 10-bit NVIDIA graphics card. The advantage of this monitor is that it can be calibrated to give a linear response in luminance and allow for resampling of the gamma curve to achieve more test-levels in the low contrast region and, hence, more accurate contrast sensitivity measurements. However, the monitor has a limited spatial resolution and maximum luminance.

## 8. University of Houston binocular adaptive optics visual simulator

### 8.1 Description of the system

**Fig. 13.**
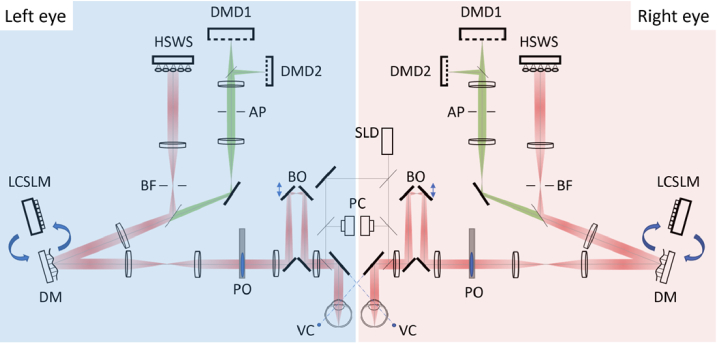
Schematic optical layout of University of Houston Binocular Adaptive Optics Visual Simulator. DM: Deformable Mirror, LCSLM: Liquid Crystal Spatial Light Modulator, HSWS: Hartmann-Shack Wavefront Sensor, SLD: Superluminescent Laser Diode, DMD: Digital Micromirror Device, VC: Vergence Control, BD: Badal Optometer, PC: Pupil Camera, PO: Phoropter, AP: Artificial Pupil, BF: Binocular Fusion lock

Figure 13 shows the schematic optical layout of the University of Houston Binocular Adaptive Optics Visual Simulator. The system consists of two identical monocular AO systems that have evolved from the original large stroke monocular AO system [115]. Each monocular system can be operated independently or synchronized by a single master computer as a binocular system. Below is a summary of the main components and unique capabilities of the system.

Main components: (1) HSWS: High-resolution Hartmann-Shack wavefront sensor (sampling resolution:133 µm, up to 30 frames/second); (2) DM: 97 actuators deformable mirror (Alpao, France) with large stroke (86 µm wavefront tip/tilt), stable closed-loop control with bandwidth up to 1.38 kHz; (3) LCSLM: Liquid-Crystal Spatial Light Modulator (Jasper Display, Taiwan), 1920 × 1080 resolution, 6.4 µm pixel pitch, amplitude/phase modulation for 430-750 nm, HDMI input; (4) DMD: Digital Micromirror Display (TI, USA), 1920 × 1080 resolution, 5.4 µm mirror pitch, HDMI input, refresh rate up to 120 Hz; (5) SLD: Superluminescent Laser Diode (Inphenix, USA), 35 nm broadband @ λ= 840 nm. Unique features of the system: (1) Monocular and binocular optical and visual testing; (2) Custom AO software: full, partial, and selective correction, aberration induction, automatic DM calibration, automatic blink detection; (3) Visual psychophysics: visual acuity, contrast sensitivity, contrast matching, natural image quality assessment, dynamic video display, stereo performance, binocular rivalry, motion detection, vision testing synchronized with AO control, (4) Motorized vergence control: binocular accommodation, virtual reality experiment; (5) Badal Optometer: motorized adjustment of the subjective best focus, target distance control without changing magnification; (6) Phoropter: pre-compensation for large sphero-cylindrical refractive errors; (7) Pupil size control: motorized artificial pupil to control static and dynamic changes in pupil diameter; (8) Peripheral vision testing with off-axis fixation

### 8.2 Key application

*The Limits of Human Vision – Long-term Neural Adaptation to Ocular Aberrations.* Visual functions are first influenced by the optics of the eye determining retinal image quality, and then by the neural system sampling the image and interpreting information via complicated neural processing. With the advent of ocular wavefront sensing technology, our understanding of the optical factors impacting vision has been greatly improved over the past three decades. Optical imperfections including aberrations and light scatter limit visual performance if uncorrected [115,116]. It is a relatively recent discovery that prolonged visual experience with poor optics can alter the way the neural system processes images formed on the retina [117,118]. A longstanding clinical example of this finding is that both resolution and contrast sensitivity after astigmatism correction were found to be poorer along the original cylindrical axis than the orthogonal orientation [119]. Despite the significance of the phenomenon from basic science and clinical wisdom, little is known about the mechanism underlying the effects of long-term neural adaptation to imperfect ocular optics on neural processing of images. The overarching goal of our project is to address questions about optical and neural contributions to human vision.

To study long-term neural adaptation to poor optics, we have been using a unique group of patients with keratoconus (KC), a chronic, non-inflammatory, bilateral corneal disease. This disease is characterized by localized thinning of the cornea, inducing large amounts of HOAs [120], which results in substantially degraded visual performance. This study group is ideal because (1) their visual system has been chronically exposed to optical defects for a long period of time and (2) the magnitude of the optical defects is significantly larger than normal subjects. Therefore, the effects of neural adaptation in KC patients are greater compared to normals, which renders investigation of neural factors more robust and accurate. Our previous studies found that visual performance measured in these patients after full AO aberration correction is significantly poorer than that predicted from optical theory and that in normal eyes [118]. We hypothesized that this unexplained loss in visual performance is an adverse effect of long-term neural adaptation to optical aberrations, which gradually alters the way the neural system processes images.

**Fig. 14.**
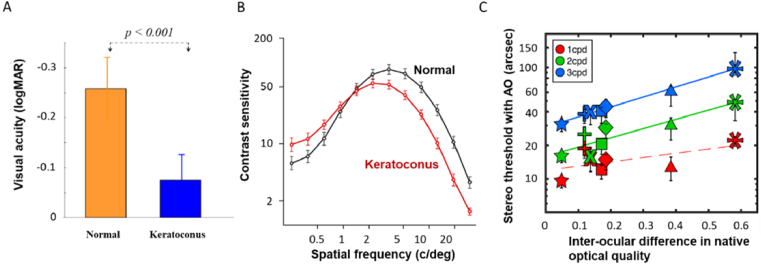
Effects of long-term neural adaptation on neural functions (A. visual acuity, B. contrast sensitivity) after full AO correction of the eye’s aberrations in normal and keratoconus subjects, and C. stereo threshold as a function of inter-ocular difference in native optical quality. Different symbols represent data from different participants.

We have identified two potential mechanisms underlying long-term neural adaptation so far; (1) adaptation to phase spectra and (2) gain adjustment of individual spatial frequency channels. Visual acuity and contrast sensitivity were measured with KC subjects who experienced their habitual aberrations and with normal subjects who experienced the equivalent KC aberrations induced by our AO [117]. Although the two groups experienced identical image quality, the KC group outperformed normal subjects when the stimuli were broadband (i.e. visual acuity) but not when they were narrowband (i.e. contrast sensitivity). This strongly suggests that the KC neural system had effectively compensated for phase incongruence induced by their habitual aberrations. This compensatory neural process through long-term adaptation to the phase spectra alleviates the detrimental effects of habitual, uncorrected optics on retinal image quality. However, this mechanism plays an adverse role in visual acuity immediately after the removal of the aberration with AO as the neural system is no longer familiar with the improved optics (Fig. 14(A)) [118]. To test the second hypothesis, we measured contrast sensitivity under fully corrected optical conditions (Fig. 14(B)). KC subjects showed atypical neural contrast sensitivity functions, revealing impaired sensitivity at high spatial frequencies (SFs) and enhanced sensitivity at low SFs compared to normal subjects [121]. These trends increased with KC severity. Notably, this effect cannot be explained by optical factors and we postulate the following. Under natural viewing, the KC visual system is exposed to an amplitude spectrum that is weighted heavily toward low SFs due to the severe optics, resulting in adaptive changes that optimize sensitivity to better match this altered diet of SFs.

*Binocular vision under modified optics.* The effects of optics on binocular vision are relatively unexplored. Binocular performance is superior to monocular performance (known as binocular summation) when each eye provides similar image quality [122]. However, when the brain combines monocular images with very different quality, binocular summation is reduced [55] and can even lead to ‘binocular inhibition’ i.e. binocular performance being worse than either monocular performance. On the other hand, our earlier work showed that the visual system can adaptively combine two monocular images when inter-ocular image quality differences are induced by mirror symmetry in the aberration profiles (e.g. horizontal coma) and orthogonal blur orientation (astigmatism) [123]. We further investigated how the eyes’ optics affect the precision of stereopsis so as to understand more about limitations imposed by post-optical mechanisms. Contrary to the previous finding [124], our work found that stereo acuity was indeed improved after correcting the HOAs, suggesting that stereo resolution is still limited by the eyes’ optics. Also, the improvement with AO was smaller in individuals who have had large inter-ocular differences in their habitual optical quality (Fig. 14(C)), indicating that past binocular experience with different monocular visual inputs affects binocular neural processing similar to our monocular findings [125]. Neural plasticity is a ubiquitous property of normal adult systems, and can also be elicited in highly aberrated eyes [126]. Given the functional importance of vision, there has been an increasing focus on training vision in individuals with typical and low vision. Future research involving advanced correction methods [127,128] will assess whether altered neural function resulting from long-term adaptation can be reversed and to what extent this can occur.

### 8.3 Current and future improvements

Although the current binocular AO system has been a powerful tool, allowing us to investigate the optical and neural contribution to human vision, there are a few technical challenges. The closed-loop bandwidth of AO is still significantly slower than typical fixational eye movements. This is due mainly to relatively slow ocular wavefront sensing due to low light levels available from the eye. A faster and high-sensitivity imaging sensor combined with computer processing power and a wavefront corrector with more degrees of freedom (i.e. actuators) will further improve our ability to achieve targeted optical quality over a long period of time.

## 9. Future trends and challenges in adaptive optics visual simulation

Since the first demonstration 25 years ago, Adaptive Optics Visual Simulators have incorporated new active elements to correct and induce aberrations, and to simulate different optical corrections (refractive, segmented and diffractive optics). Some recent systems can correct not only monochromatic but also chromatic aberrations. They have increased speeds in wavefront measurement and corrections, and are even binocular. These expanded capabilities have increased the number of applications, and their clinical use. While some systems have moved into commercial devices and started to be used in the daily clinical practice, overcoming some technical challenges will open up new research, development and clinical application opportunities. Desirable features of the next generation of Adaptive Optics Visual Simulators include aberration measurement and correction in a small footprint, potentially wearable device, wide and open-view visual field stimulation, increased speed and integration with other biometry imaging devices. Envisioned solutions to these issues include: (1) see-through wavefront correctors instead of mirror-based solutions, including deformable lenses and transmission spatial light modulators with increased spatial and temporal resolutions over state-of-the-art, (2) new generation of optical elements, in line with those being developed in AR/VR technologies, (3) eye-tracking system, (4) faster response and higher-sensitivity imaging sensors, faster control and processing algorithms and more powerful computer architectures and (5) integrated photonics and optical devices.

## Data Availability

Data underlying the results presented in this paper are not publicly available at this time but may be obtained from the authors upon reasonable request.
